# Defining three dimensional chromatin structures of pediatric and adolescent B cells using primary B cell and EBV-immortalized B cell reference genomes

**DOI:** 10.1186/s12920-025-02166-9

**Published:** 2025-05-28

**Authors:** Kaiyu Jiang, Yao Fu, Jennifer A. Kelly, Patrick M. Gaffney, Lucy C. Holmes, James N. Jarvis

**Affiliations:** 1 Department of Pediatrics, Clinical and Translational Research Center, Jacobs School of Medicine and Biomedical Sciences, University of Buffalo, 875 Ellicott St, Buffalo, NY 14203 USA; 2 University of Washington Rheumatology Research, 750 Republican St, Seattle, WA E52398109 USA; 3 Genes and Human Disease Research Program, Oklahoma Medical Research Foundation, Oklahoma City, OK 73104 USA; 4 Genetics, Genomics and Bioinformatics Program, Jacobs School of Medicine and Biomedical Sciences, University of Buffalo, 875 Ellicott St, Buffalo, NY 14203 USA

**Keywords:** B cells, Chromatin accessibility, Chromatin conformation, Pediatric autoimmune disease, Juvenile arthritis, Systemic lupus, Type 1 diabetes

## Abstract

**Background/Purpose:**

Knowledge of the 3D genome is essential to elucidate genetic mechanisms driving autoimmune diseases. The 3D genome is distinct for each cell type, and it is uncertain whether cell lines faithfully recapitulate the 3D architecture of primary human cells or whether developmental aspects of the pediatric immune system require use of pediatric samples. We undertook a systematic analysis of B cells and B cell lines to compare 3D genomic features encompassing risk loci for juvenile idiopathic arthritis (JIA), systemic lupus (SLE), and type 1 diabetes (T1D).

**Methods:**

We isolated B cells from four healthy individuals, ages 9–17. HiChIP was performed using a CTCF antibody, and CTCF peaks were called within each sample separately. Peaks observed in all four samples were identified. CTCF loops were called within the pediatric samples using three CTCF peak datasets: 1) self-called CTCF consensus peaks called within the pediatric samples, 2) ENCODE’s publicly available GM12878 CTCF ChIP-seq peaks, and 3) ENCODE’s primary B cell CTCF ChIP-seq peaks from two adult females. Differential looping was assessed within the pediatric samples and each of the three peak datasets.

**Results:**

The number of consensus peaks called in the pediatric samples was similar to that identified in ENCODE’s GM12878 and primary B cell datasets. We observed < 1% of loops that demonstrated significantly differential looping between peaks called within the pediatric samples themselves and when called using ENCODE GM12878 peaks. Significant looping differences were even fewer when comparing loops of the pediatric called peaks to those of the ENCODE primary B cell peaks. When querying loops found in juvenile idiopathic arthritis, type 1 diabetes, or systemic lupus erythematosus risk haplotypes, we observed significant differences in only 2.2%, 1.0%, and 1.3% loops, respectively, when comparing peaks called within the pediatric samples and ENCODE GM12878 dataset. The differences were even less apparent when comparing loops called with the pediatric vs ENCODE adult primary B cell peak datasets.

**Conclusion:**

The 3D chromatin architecture in B cells is similar across pediatric, adult, and EBV-transformed cell lines. This conservation of 3D structure includes regions encompassing autoimmune risk haplotypes. Thus, even for pediatric autoimmune diseases, publicly available adult B cell and cell line datasets may be sufficient for assessing effects exerted in the 3D genomic space.

**Supplementary Information:**

The online version contains supplementary material available at 10.1186/s12920-025-02166-9.

## Introduction

B cells are known to play an important role in the pathobiology of a wide range of autoimmune diseases that can have or exclusively have a pediatric onset [1–5]. However, a serious limitation to our study of these cells in pediatric disease is the relatively small number of cells in the circulation (B cells represent about 20% of the circulating lymphocytes) and the limited volume of blood that can be acquired from children. Further complicating the problem are the practical and ethical considerations of acquiring blood from healthy children to isolate B cells that can be used as control or reference data.

After the completion of the mapping of the human genome, the National Institutes of Health embarked on an ambitious effort to characterize the non-coding, functional elements (i.e., those elements composing 98% of the genome) in medically-important organisms, cell lines, and primary human cells. These two projects, the Encyclopedia of (Functional) DNA Elements (ENCODE) [6] and Roadmap Epigenomics [7] have provided investigators with a treasure trove of information that can be used to inform our understanding of data derived from patients with a broad range of diseases. Unfortunately, these genomic reference data sets are lacking data from children, although fetal and cord blood samples are represented in the collection. This data gap has forced investigators of pediatric diseases to generate their own genomic data sets on an ad hoc basis [8], often at considerable expense. This barrier limits the number of patients who can be studied in research aimed at understanding genetic/genomic mechanisms of disease, as the costs of acquiring samples from both patients and controls as well as the procedures themselves (e.g., sequencing costs) must be included in limited research budgets. The perceived need for acquiring reference samples from healthy children, rather than using ENCODE and Roadmap Epigenomics reference sets, represents on “hidden tax” on pediatric investigators that may not be placed on investigators of adult diseases, who can use these reference data without any concerns about their suitability.

Despite the concerns about using adult data or human cell lines for pediatric genomic research, the differences between adult and pediatric peripheral blood cells have not been examined in detail. Zhao et al. demonstrate differences in the genomic locations of CpG methylation sites when they compared adult and pediatric CD4 + cells [9]. Beyond this single study, however, little else is known. Here, we compare the three-dimensional chromatin structures of adult and pediatric/adolescent peripheral blood B cells and with the commonly used B cell line, GM12878. We demonstrate that the CTCF-anchored three-dimensional loop structures within these different cell types differ at fewer than 1% of shared locations across the genome. Furthermore, we show that these small differences are no greater within the regions that harbor SNPs tagging the risk loci for juvenile idiopathic arthritis (JIA), systemic lupus erythematosus (SLE), and type 1 diabetes (T1D) than in the broader genome.

## Materials and methods

### Study subjects

Healthy children/adolescents were recruited under a University at Buffalo IRB-approved protocol **(#**MODCR00007255**)** from the University at Buffalo Medical Doctors Pediatric Clinic. Four subjects were included in this study, 3 boys (ages 9, 16, and 16) and 1 girl (age 17). Informed consent was obtained from the parents of the children/adolescents providing samples, and assent was obtained from each of the participating individuals. The study was carried out in accordance with the Federal Policy for Protection of Human Subjects, which is based on the Belmont Report of the U.S. National Commission for the Protection of Human Subjects of Biomedical and Behavioral Research.

### Isolation and Preparation of B cells

Whole blood (total 24 ml) was drawn into 8 mL citrated Cell Preparation Tubes (Becton Dickinson, Franklin Lakes, NJ, USA). Specimen processing was started within one hour from the time the specimens were drawn. Peripheral blood mononuclear cells were separated from granulocytes and red blood cells by density-gradient centrifugation. B cells were purified from PBMC by negative selection using the StemSep™ Human B Cell Enrichment Kit (STEMCELL Technologies Inc., Vancouver, Canada) following the manufacturers’ instructions.

### CTCF HiChIP

B cells (~ 2 × 10^6^ each sample) were fixed in 1% formaldehyde at room temperature for 10 min. The fixation reaction was quenched by adding glycine (final concentration of 125 mM) and incubated for 5 min at room temperature with rotation. Cells were pelleted at 500xg at room temperature for 5 min. Cell pellet were rinsed once using 1 ml PBS. HiChIP assays were performed as described in Mumbach et al., 2016 with some modifications. Briefly, nuclei were isolated from fixed B cells and subjected to in situ digestion using 200U MboI (NEB, R0147M) for 4 h at 37 ºC. The restriction fragment overhangs were filled and labeled with dCTP, dGTP, dTTP and biotin-dATP using Klenow DNA polymerase I (NEB M0212L), followed by in situ proximity ligation at room temperature overnight. The chromatin was fragmented by 2-min sonication with the Covaris E220 system, then immunoprecipitated using CTCF antibody (Cell Signaling, 3418S) at 4ºC overnight. DNA was purified by Zymo DNA Clean & Concentrator kit (Zymo, D4003). Streptavidin C1 beads were used to capture biotin-labeled DNA fragments. The sequencing libraries were generated on the streptavidin C1 beads using Illumina Tagment DNA Enzyme and Buffer kit (Illumina, 20,034,198) and run on the Illumina Novaseq S4 flowcell.

### Statistical analyses

#### Initial QC and read processing

Adapters were removed from sequenced fastq files by fastp (options: detect_adapter_for_pe -l 50 -x –g) [10]. Samples 3 (246 M reads) and 6 (263 M reads) were downsampled to 200 M reads using seqtk for downstream processing (https://github.com/lh3/seqtk). Reads were aligned to the human genome using Bowtie2’s *H. sapiens* GRCh38 no alt analysis set with a mapQ > 10 and the following options: for global alignment: –very-sensitive -L 30 –score-min L,−0.6,−0.2 –end-to-end –reorder, and for local alignment: –very-sensitive -L 20 –score-min L,−0.6,−0.2 –end-to-end –reorder [11, 12]. Reads were then processed using a MboI hg38 restriction fragment bed file and the HiC-Pro analysis pipeline (see Supplementary Table 1 and Supplementary Fig. 1 for QC stats) [13]. CTCF HiChIP read tracks were RPCG normalized for viewing.

#### Pediatric/adolescent sample CTCF peak calling

 HiChIP peak sets were called within each sample separately using MACS2 with defaults, a genome size = 2,913,022,398, and FDR q = 0.01 [14]. Peaks were evaluated for known ENCODE blacklisted regions, but no overlap was found. A consensus peak set was generated from individual peak profiles using DiffBind [15] for reproducible peaks observed in all four samples.

#### CTCF loop calling

CTCF loops were called within the pediatric samples using HiChipper [16] using three CTCF peak datasets: 1) CTCF peak set called within the pediatric samples using MACS2 described in 2.4.2. above, 2) the ENCODE GM12878 CTCF ChIP-seq peak set (reference epigenome ENCSR000 AKB, GRCh38 accession ENCFF017XLW processed 2020–10-01, and 3) the ENCODE primary B cell CTCF ChIP-seq peak set from two adult females (reference epigenome ENCSR682 AXR, GRCh38 accession ENCFF919YTB processed 2020–10–20). Differential loop calling was determined using DiffLoop [17] with a Mango q < 0.01. Anchors within 500 bp were merged into single peaks. Loops were then restricted to those called within 2 or more samples of each comparison group and paired end tag sequencing (PETS) ≥ 2 in both comparison groups, similar to previously reports [17, 18].

#### Gene set enrichment analysis (GSEA)

Loop anchors were annotated to gene transcript start sites (TSS) within 1 M bp using the GenomicRanges library in R. A GSEA was then performed for the 500 genes with distances to TSS < 45 kb using the MsigDB hallmark gene sets [19, 20]. An FDR < 0.05 was used to determine pathways enriched for genes.

#### Evaluation of TAD structures and the genes within them

Contact maps for viewing in Juicebox were created for the pediatric samples using HiC-Pro’s hicpro2juicebox.sh utility [21]. Topologically associated domains (TADS) were determined visually and analyzed for *ENRAP2, IRF1*, and *IL6* JIA risk haplotypes as previously described [22].

## Results

### CTCF loop structures within pediatric/adolescent primary B cells are similar to those in ENCODE GM12878 and adult primary B cell datasets

**Fig. 1:**
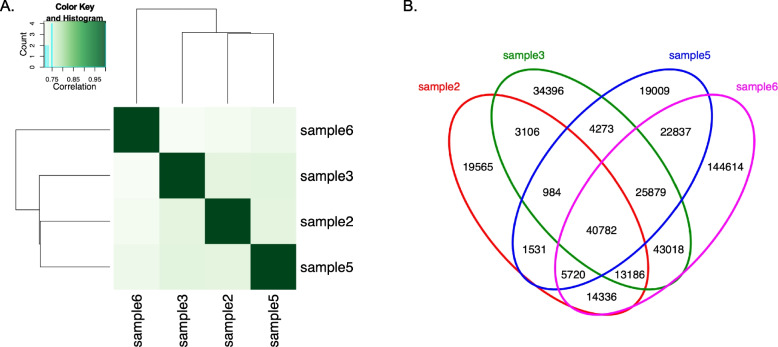
CTCF peaks in pediatric primary B cells**. A** Correlation heat map of CTCF peaks in four pediatric samples as called by MACS2. The four samples show strong reproducibility (r.^2^ > 0.7) of peaks amongst them. **B** Venn diagram of shared CTCF peaks between the four pediatric samples as determined by DiffBind. A total of 40,782 peaks are observed in all four samples (consensus peak set)

A total of 393,236 CTCF peaks (range: 101,230–311,719) were called in the four pediatric/adolescent samples with strong correlation (r^2^ > 0.7) and an average peak size of 257.08 bp (Fig. 1A, Supplementary Table 2). A set of 40,782 peaks (consensus peak set) were observed in all four samples (Fig. 1B, Supplementary Table 3). This number of consensus peaks called in the pediatric/adolescent samples was similar in size to the number of CTCF peaks identified in the ENCODE GM12878 dataset (*n* = 41,017), while a bit smaller than the primary B cell dataset (*n* = 53,854), and had average and median peak sizes of 463.60 bp and 397.0 bp (compared to GM12878: 309 bp and 356 bp; ENCODE primary B cell dataset: 459 bp and 480 bp).

**Fig. 2:**
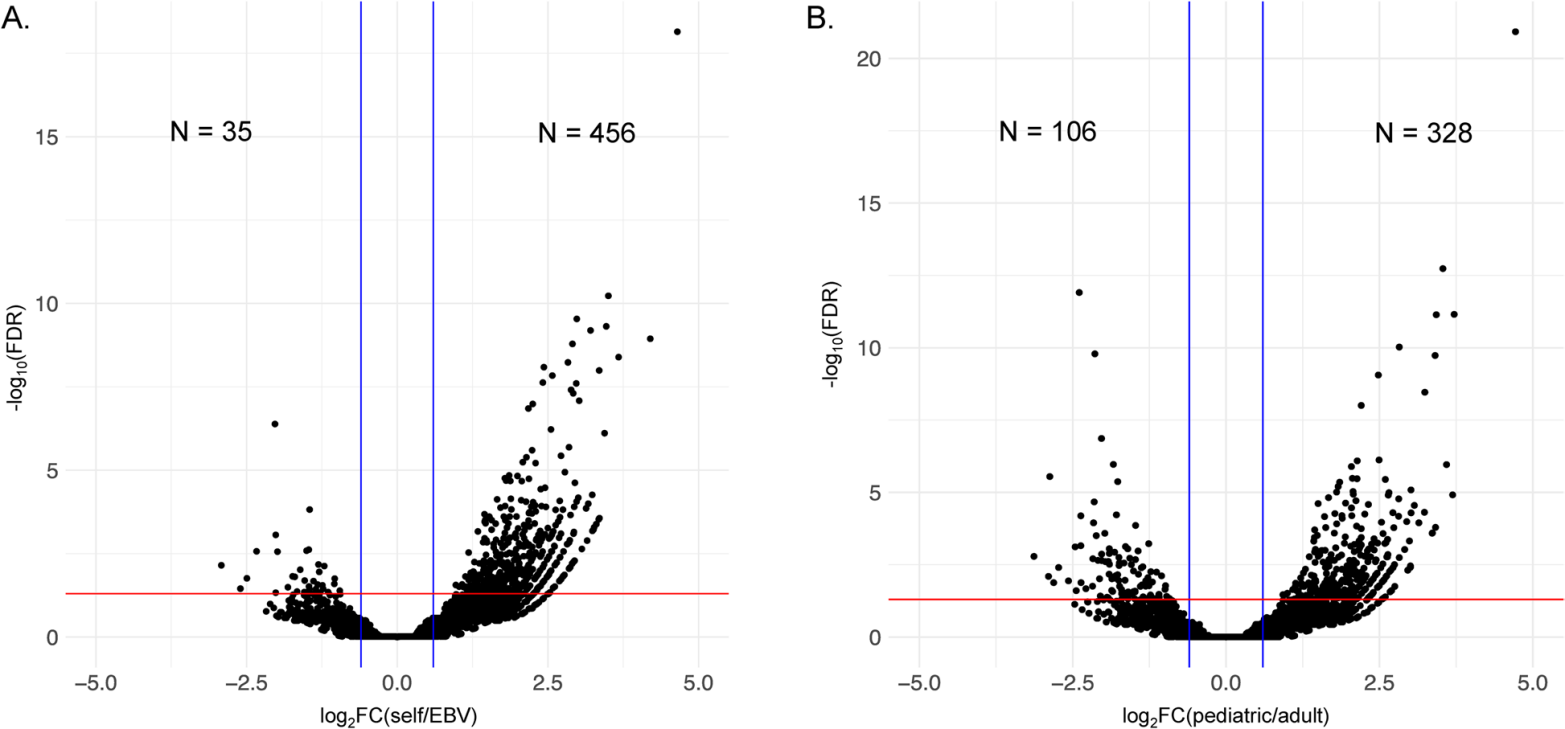
CTCF loop differential analysis volcano plots of in pediatric samples**. A** Differential analysis of loops using self-called pediatric CTCF peaks compared to ENCODE GM12878 CTCF peaks. **B** Differential analysis of loops using self-called pediatric CTCF peaks compared to ENCODE adult primary B cell peaks. Vertical blue lines represent a FC ≥ 1.5 or ≤ −1.5 and red horizontal line represents an FDR q ≤ 0.05. Log_2_FC is plotted on the x-axis and –log_10_(FDR) is plotted on the y-axis. Numbers representing significantly higher PETs in self called peaks are to the right of the graph and those with higher PETs in ENCODE datasets to the left of the graph

When evaluating loops generated in the pediatric/adolescent datasets using their own CTCF peaks and using the GM12878 CTCF peaks, a total of 61,860 shared CTCF loops (*n* = 181,316 prior to filtering) were called by DiffLoop for differential analysis (Supplementary Table 4). Only 0.8% (*n* = 491) of loops were significantly different (FDR < 0.05) between the two groups, of which 456 (93%) had significantly more PETs in the pediatric called peaks compared to the GM12878 peaks (Fig. 2A). The most significantly differential looping is on chromosome 22 between loop anchors at chr22:22,679,600–22688604 and chr22:22,691,237–22,693,712 with stronger loops observed in the samples when called with the pediatric peaks (Supplementary Table 4; Supplementary Fig. 2). This suggests that the overall 3D chromatin architecture in primary B cells collected from pediatric/adolescent samples is similar to that of the publicly available GM12878 cell line dataset and that the cell line data should be sufficient for assessing effects exerted in the 3D genomic space of pediatric samples, at least with respect to interaction that occur within the confines of CTCF-anchored topologically associated domains. The anchors that were involved in the 491 differential loops are near 661 genes (Supplementary Table 4). Performing a GSEA showed that these genes are enriched (q < 0.05) in the following pathways: TNFA signaling via NFKB (q = 3.48E-5), allograft rejection (q = 1.07E-4), estrogen response early (q = 3.0E-4), myogenesis (q = 3.0E-4), and others (Supplementary Tables 5 & 6). The majority of genes (*n* = 489, 74%) were involved in only one differential loop (Supplementary Fig. 3a). Several genes, however, were involved in multiple differential looping events, with the EH domain containing 1 gene (*EHD1*) on chromosome 11 being involved with the highest number of differential looping events: eight. However, this gene has not yet been associated with any trait or phenotype (https://www.ebi.ac.uk/gwas/search?query=EHD1). We provide a table of the genes involved in differential looping events in Supplementary Table 7.

We next evaluated the ENCODE adult primary B cell CTCF peak dataset to see how similar it was to the peaks called in the pediatric/adolescent primary B cell dataset. We observed fewer significant looping differences when comparing these two datasets than we observed when comparing primary pediatric and GM12878 cells. A total of 70,909 (n = 195,221 prior to filtering) CTCF loops were identified for differential analysis, with 434 (0.61%) of the loops showing significant differences (FDR < 0.05) between the two groups (Fig. 2B, Supplementary Table 8). A total of 328 (75.6%) loops had significantly more PETs in the pediatric/adolescent consensus peaks compared to the ENCODE adult peaks, while 106 loops had significantly more PETS in the ENCODE adult peaks. The 151 genes near anchors with more PETs in the ENCODE adult peak loops were enriched for the following pathways: myogenesis (q = 0.022), MYC targets version 2 (q = 0.022), coagulation (q = 0.022), apoptosis (q = 0.029), *IL2-STAT5* signaling (q = 0.042), and hypoxia (q = 0.042) (Supplementary Tables 9 A & 10 A). The 459 genes near anchors with more PETs in the pediatric peak loops were enriched for the following pathways: hypoxia (q = 9.58E-5), *TNFA* signaling via *NFKB* (q = 2.92E-4), apical junction (q = 3.27E-3), *KRAS* signaling DN (q = 3.27E-3), *MTORC1* signaling (q = 3.27E-3), and others (Supplementary Tables 9B & 10B). Looking at the 591 total genes annotated near differential loops in this analysis, 455 (77%) were involved in only one differential loop (Supplementary Fig. 3B), and the greatest number of loops attributed to any gene was five.

### Loop structures within and subsuming the JIA, T1D, and SLE risk haplotypes are not significantly different when using peaks called within pediatric primary B cells compared to publicly available datasets

We next sought to determine whether regions conferring risk for childhood-onset autoimmune diseases showed any notable differences in loop structures compared to the global analysis. We queried the risk regions for three autoimmune diseases in which B cells play an important role in pathobiology: JIA [5], T1D [1], and SLE [2]. We first compared loops generated in the pediatric samples using the self-called MACS2 peaks to loops called in the pediatric samples using the ENCODE GM12878 B cell line peak dataset. A total of 313 CTCF-anchored loops (*n* = 1,119 prior to QC filtering) were shared in 29 regions conferring risk for JIA (Supplementary Table 11). Only seven loops (2.2%) exhibited differential looping (FDR < 0.05) between the two groups, with all loops having more PETs in the pediatric called peaks. These loops were located in the following six JIA risk regions: *JAK1* (chr1:64,661,309–64851277), *AFF3-LONRF2* (chr2:100,208,154–100273161, and chr2:100,208,154–100273161), *WDFY4* (chr10:48,581,280–48686719), *ZFP36L1* (chr14:68,790,795–68851245), *TYK2* (chr19:10,363,179–10430870), and *IL2RB* (chr22:37,139,351–37,210,641). T1D risk haplotypes generated 1,350 shared loops (*n* = 4,423 prior to QC filtering) (Supplementary Table 12). A total of 13 loops (1.0%) on 10 T1D haplotypes displayed differential looping, with all loops having more PETs in the pediatric called peaks. The most significantly differential loops were located on *IL2RB-C1QTNF6* haplotype (chr22:37,139,351–37,210,641 and chr22:37,154,012–37226586) Supplementary Table 12). When looking at loops in regions conferring risk for SLE, a total of 2,398 loops (*n* = 7,532 prior to filtering) were shared (Supplementary Table 13). Only 31 loops (1.3%) were significantly different between the two groups, with all loops having more PETs in the pediatric called peaks. These 31 loops involved 22 lupus haplotypes, with *ETV3-FCRL5, BLK,* and *IRF8* haplotypes containing more than one significantly differential loop (Supplementary Table 13).

We then compared loops generated in the pediatric samples using the self-called MACS2 peaks to loops called in the pediatric samples using the ENCODE adult primary B cell peak dataset and observed even fewer differences between the two peak datasets. A total of 388 CTCF-anchored loops (*n* = 1,105 prior to QC filtering) were shared in 30 regions conferring risk for JIA (Supplementary Table 14). Only five loops (1.3%) exhibited differential looping between the two groups. Two loops had significantly fewer PETs when called using the pediatric self-called peaks compared to the adult primary B cell peaks (*ATP8B2-IL6R:* chr1:154,298,690–154361733, and *ICAM3-TYK2* (chr19:10,243,816–10447681), while the remaining three differential loops had more PETs when called using the pediatric self-called peaks (all of which also had significantly more PETs in the self-called peaks when compared against the GM12878 peaks): *WDFY4* (chr10:48,581,280–48686719), *ZFP36L1* (chr14:68,790,795–68851245), and *TYK2* (chr19:10,363,179–10430870). T1D risk haplotypes generated 1,679 shared loops (*n* = 4,764 prior to QC filtering) (Supplementary Table 15). A total of 10 loops (0.6%) on 9 T1D haplotypes displayed differential looping. Again, the two loops in the regions of *ATP8B2-IL6R* (chr1:154,298,690–154361733) and *ICAM3-TYK2* (chr19:10,243,816–10447681) had significantly fewer PETs in the self-called pediatric peaks compared to the adult B cell peaks while the remaining eight loops had significantly more PETs in the pediatric self-called peaks. When looking at loops in regions conferring risk for SLE, a total of 2,896 loops (*n* = 7,808 prior to filtering) were shared (Supplementary Table 16). Only 27 loops (0.9%) on 15 lupus haplotypes were significantly different between the two groups; four loops had significantly fewer PETs in the pediatric self-called peaks compared to the adult primary B cell peaks (*PARP11:* chr12:3,857,402–3990865, *PLD2:* chr17:4,705,955–4930243, *ARID3 A:* chr19:834,045–1010097, and *TYK2:* chr19:10,243,816–10447681), and the remaining 23 loops had significantly more PETs in the pediatric self-called peaks.

## Discussion

As a group, the rheumatic diseases of childhood are among the most common chronic disease conditions in children [23, 24]. B cells are known to play important roles in the pathobiology of these conditions [4, 5, 25], as well as other pediatric autoimmune diseases [3, 26]. Furthermore, strong genetic risk associations have been identified for a broad spectrum of autoimmune diseases of pediatric onset. Because, for so many autoimmune diseases [27] including JIA [8, 28] and SLE [29] genetic risk is exerted most strongly by variants within the non-coding genome, elucidating genetic mechanisms will invariably require a broader understanding of the 3D genome of disease-relevant cells [8], with comparisons of cells from both healthy and affected individuals. The need for such comparisons was one of the important drivers of the ENCODE [6], Roadmap Epigenomics [7], and Blueprint Epigenomics [30] projects, which now provide invaluable reference data for the relevant epigenetic and functional features of the non-coding genomes of multiple tissues and cells. Whether these data sets can be used to interrogate genetic mechanisms in childhood onset diseases like JIA is unknown. Until now, the standard has been for investigators of these diseases to generate their own reference data from healthy children [8, 31, 32], something that is difficult and costly to do. The field of pediatric autoimmunity would benefit greatly if it could be ascertained that existing reference data sets are applicable, especially at autoimmune risk loci. As far as we know, this is the first attempt to compare the 3D chromatin structures of the commonly B cell line, GM12878, and primary human B cells (either adult of pediatric/adolescent) with respect to 3D chromatin architecture around autoimmune risk loci.

In this study, we used CTCF HiChIP to define the 3D architecture of pediatric B cells and compared findings with existing data sets derived from adult B cells and B cell lines. We find only small differences in the overall chromatin architecture when we compare consensus peaks between primary pediatric B cells and adult B cells and B cell lines. This contrasts with our earlier comparisons across cell lines, where the 3D chromatin structures (assessed by HiC) that encompass the JIA risk loci differ substantially and even encompass different potential target genes [22]. Furthermore, when we specifically examined the regions known to confer genetic risk for three autoimmune diseases (JIA, SLE, and T1D), we find no specific features that make the 3D chromatin architecture of these regions idiosyncratic. Therefore, for studies examining genetic mechanisms influencing B cells and driving these diseases, the existing reference data sets are, in most instances, likely to be suitable. Our findings therefore mirror those of Ray et al. [33] who found that, for most studies aimed at elucidating genetic mechanisms in autoimmunity, the chromatin architecture of commonly used cell lines is sufficiently faithful to primary cells in most instances to allow their use. We would caution, as do Ray et al., that investigators carefully examine their locus of interest to be sure that the cell or cell line faithfully recapitulates primary cells in that region before undertaking mechanistic studies with CRISPRi to define, for example, the targets of regulatory elements (which are determined by 3D chromatin architecture) or any of the multiple genome editing approaches to define the effects of specific SNPs on those regulatory elements.

The discovery that much of the genetic risk for autoimmune diseases likely resides within the non-coding regulatory regions [8, 27] highlighted the importance of understanding the 3D structure of chromatin in disease-relevant cells. Regulatory structures such as enhancers may not regulate the most proximal gene in terms of linear genomic distance. Indeed, enhancers harboring disease-driving single nucleotide polymorphisms (SNPs) lying on autoimmune haplotypes may regulate genes that are not actually on the disease haplotype [34]. However, enhancers and other regulatory elements typically regulate genes within the same CTCF-anchored chromatin loop or topologically associated domain [35]. For example, in a genome-wide screening of 5,920 candidate enhancers, Gasperini and colleagues found that > 70% of enhancers regulate genes within the same CTCF-anchored TAD [35]. Thus, identifying target genes, i.e., the genes whose expression levels/function are influenced by the disease-driving SNPs on the autoimmune disease risk haplotypes, requires that one identify the relevant physical interactions between regulatory elements such as enhancers and the promoters of the candidate target genes. While relevant interactions might also be identified using H3 K27ac ChIPseq-HiChP (where results might show greater differences between the different cells/cell line used here), the arrival of newer methods to refine our knowledge of the 3D genome, such as MicroC, (which provides resolution at the nucleosome level [36]) may greatly accelerate our ability to clarify genetic mechanisms by identifying target genes.

One limitation of this study is the fact that our pediatric sample consists of only one pre-adolescent, with the other B cell samples being taken from adolescents. It is possible that samples from younger, pre-pubertal children might display differences not seen in this mostly adolescent sample. The demographics of our sample reflect the challenges of obtaining peripheral blood cells from younger, healthy children. It is important to note, however, that ENCODE and Roadmap Epigenomics data, to which our samples were compared, used the same number of replicates to generate the consensus peaks, which are what we aimed to identify in this study. Furthermore, this is the only systemic analysis of B cell 3D chromatin structures encompassing the risk loci of important autoimmune disease such as systemic lupus and T1D. Given the small differences we observed in the primary B cell sample sets we studied here, there is reason to be confident that, for most queries into genetic effects exerted on the 3D genome, the data we have generated here will provide a suitable reference set.

In conclusion, we demonstrate that the 3D chromatin architecture of primary pediatric B cells differs little from that of adult B cells or B cell lines. Thus, for most studies at aim clarifying genetic mechanisms influencing gene expression in B cells, existing cell lines and reference data sets are likely to provide valid and relevant information. Furthermore, the data we have generated here will serve as a new and valuable reference data set for those regions where there may be identifiable and important differences between adult and pediatric B cells.

## Supplementary Information

Supplementary Material 1.

Supplementary Material 2.

Supplementary Material 3.

## Data Availability

Sequencing data from the  CTCF ChIPseq and Hi-ChIP experiments have been deposited on the Gene Expression Omnibus, accession code #GSE242557.

## Abbreviations

ChIP-seq: Chromatin immunoprecipitation-sequencing
ENCODE: Encyclopedia of Functional DNA elements
JIA: Juvenile idiopathic arthritis
PETS: Paired end tag sequencing
SLE: Systemic lupus erythematosus
T1D: Type 1 diabetes
