# Supplementary material for: Defining three dimensional chromatin structures of pediatric and adolescent B cells using primary B cell and EBV-immortalized B cell reference genomes
Source: BMC Med Genomics. 2025 May 28;18:97. doi: 10.1186/s12920-025-02166-9 (PMC12117681; doi:10.1186/s12920-025-02166-9)

Supplementary Material

Supplementary Figure 1. Most significant differential looping event between pediatric samples using self-called CTCF peaks or GM12878 CTCF peaks (chr22). Loops are shown for each pediatric sample with self-called peaks on top and GM12878 CTCF peaks on the bottom. The position of both anchors are shown and the loops attributed to the anchors are in the black square.

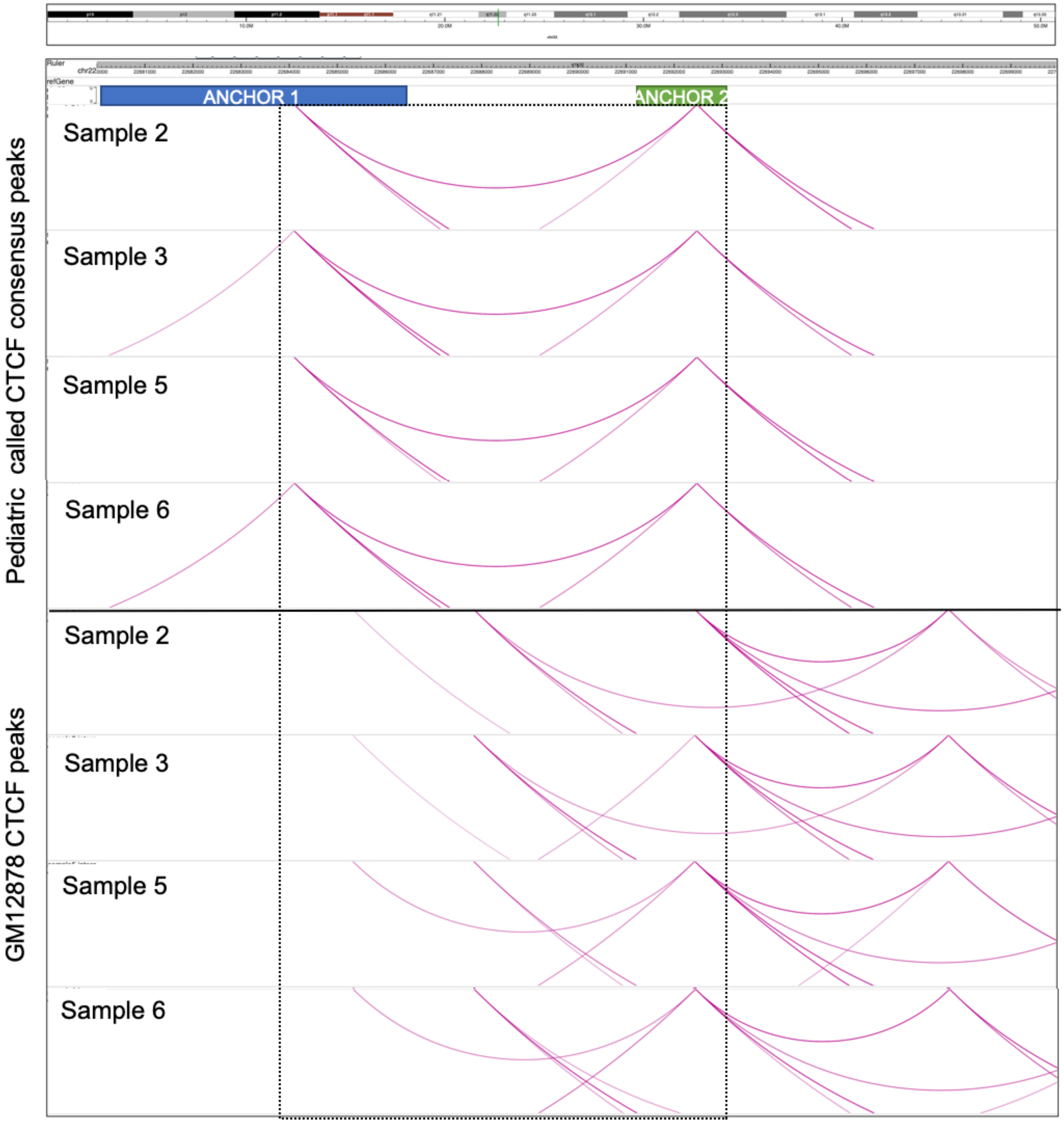

Supplementary Figure 2. Number of significantly differential loops per gene. A. Pediatric primary B cell self-called CTCF consensus peaks and ENCODE GM12878 CTCF peaks. B. Pediatric primary B cell self-called CTCF consensus peaks and ENCODE primary B cell adult CTCF peaks.

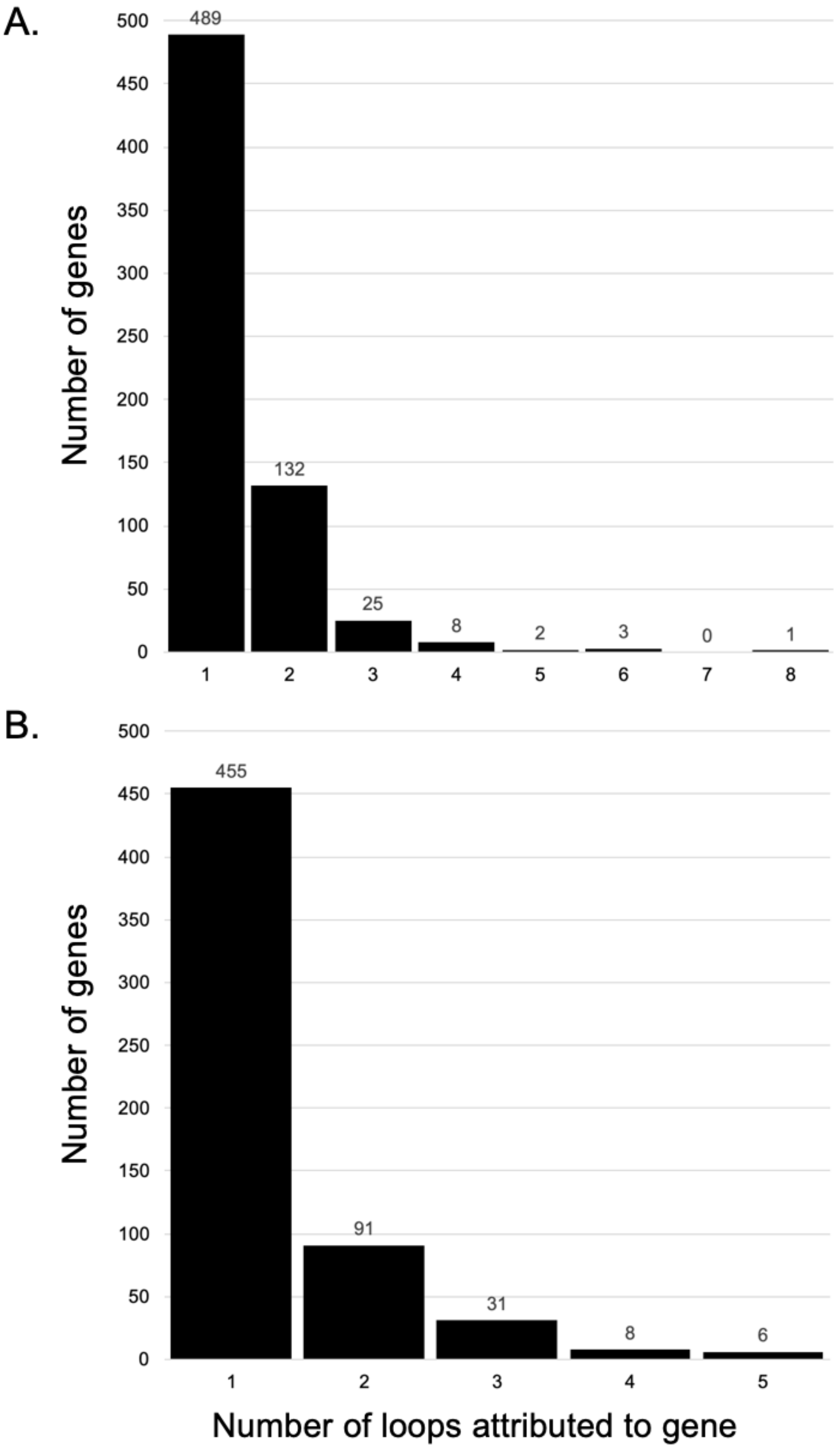

Supplementary Figure 3. Global enrichment of reads around CTCF Hi-ChIP peaks in primary B cells collected from four pediatric samples. Distance from the peak center is plotted on the x-axis and fold coverage change based on average coverage is plotted on the y-axis. The fold coverage change based on average coverage is calculated by taking the mean HiChIP/base coverage at each base within 1kb of ChIp-seq peak center, then dividing the # read pairs at each base pair by the mean HiChIP/base coverage, and finally calculating the mean of the coverage fold change across all ChIP peak centers.

Sample 2

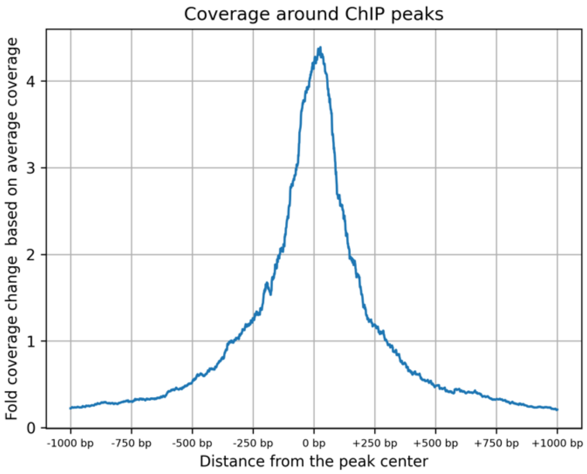

Sample 3

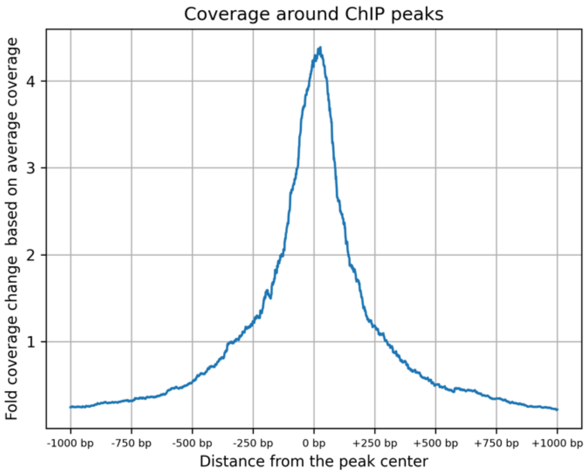

Sample 5

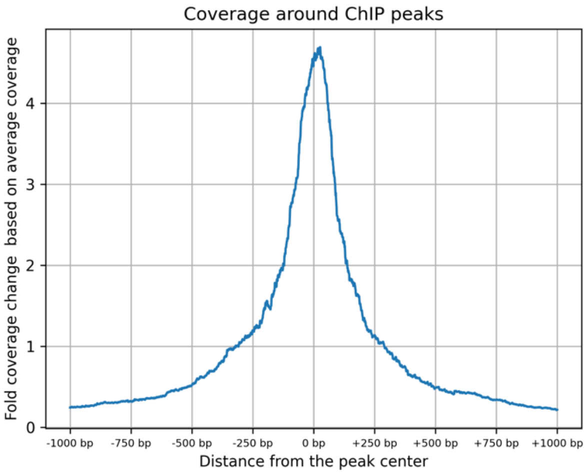

Sample 6

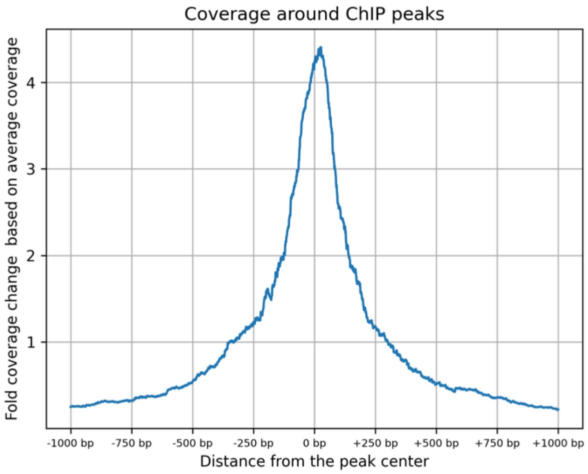

Supplement: Supplementary file 1 — Supplementary Material 1. [file 12920_2025_2166_MOESM1_ESM.pdf]
